# Gender, Estrogen, and Obliterative Lesions in the Lung

**DOI:** 10.1155/2017/8475701

**Published:** 2017-04-02

**Authors:** Hamza Assaggaf, Quentin Felty

**Affiliations:** Department of Environmental & Occupational Health, Florida International University, Miami, FL, USA

## Abstract

Gender has been shown to impact the prevalence of several lung diseases such as cancer, asthma, chronic obstructive pulmonary disease, and pulmonary arterial hypertension (PAH). Controversy over the protective effects of estrogen on the cardiopulmonary system should be of no surprise as clinical trials of hormone replacement therapy have failed to show benefits observed in experimental models. Potential confounders to explain these inconsistent estrogenic effects include the dose, cellular context, and systemic versus local tissue levels of estrogen. Idiopathic PAH is disproportionately found to be up to 4 times more common in females than in males; however, estrogen levels cannot explain why males develop PAH sooner and have poorer survival. Since the sex steroid hormone 17*β*-estradiol is a mitogen, obliterative processes in the lung such as cell proliferation and migration may impact the growth of pulmonary tissue or vascular cells. We have reviewed evidence for biological differences of sex-specific lung obliterative lesions and highlighted cell context-specific effects of estrogen in the formation of vessel lumen-obliterating lesions. Based on this information, we provide a biological-based mechanism to explain the sex difference in PAH severity as well as propose a mechanism for the formation of obliterative vascular lesions by estrogens.

## 1. Introduction

Lung disease is not only responsible for more than 349,000 deaths per year in the United States but also is a chronic condition with more than 35 million Americans living with chronic lung disease according to the American Lung Association. The increased prevalence in women of certain lung diseases such as asthma, cystic fibrosis (CF), and chronic obstructive pulmonary disease (COPD) suggests that sex-specific hormones have detrimental effects on the lung [1]. The lung is a target tissue of estrogen. Since the lung expresses estrogen receptor (ER) subtypes, ER*α* and ER*β*, estrogen has been implicated as a risk factor. The controversy over whether estrogen is protective or detrimental to the cardiopulmonary system should be of no surprise as clinical trials have failed to show cardiovascular benefits from hormone therapies. The Women's Health Initiative reported that long-term use of estrogen may have increased risk of cardiovascular disease [2] while a significant increase of coronary heart disease was observed among men receiving estrogens in the Coronary Drug Project [3, 4]. Since the sex hormone 17*β*-estradiol (E2) is a mitogen, a possible explanation may be that exposure to E2 contributed to atherosclerotic lesions, which have been proposed to occur as a result of the monoclonal expansion of a mutated vascular cell [5].

The dose of estrogens reportedly used in experimental models and clinically may offer a potential explanation for the estrogen paradox. On the one hand, estrogen at low doses acts as a pro-oxidant, whereas at higher doses, it acts to suppress oxidative stress [6–10]. In order to understand the actions of estrogen in lung cells, it is important that we understand estrogen actions which we have summarized in brief. The classical paradigm of estrogen mechanism of action is through the ER which has been extensively reviewed; therefore, we have limited our discussion in this area. Estrogen supports cell growth via interaction with estrogen receptors alpha and beta (ER*α* and ER*β*) by directly binding to estrogen response elements or through nongenomic pathways. The nongenomic action of estrogen very often includes ligand-dependent activation of GPR30 at the plasma membrane and leads to the activation of signaling pathways such as ERK/MAPK, protein kinases A and C, and calcium pathways [11]. Together, these genomic and nongenomic pathways can contribute to obliterative lesions via cell proliferation. Alternatively, reactive oxygen species (ROS) generated from redox cycling of both stilbene and catechol estrogens can act as signaling messengers also that are also involved in cell growth [7, 12, 13]. We have shown that physiologically achievable E2 concentrations, corresponding to the estrogenic menstrual peak, induce formation of ROS. Importantly, the ROS produced as a result of estrogen stimulation does not require estrogen receptors, as the ER-negative cell line produces a similar amount of ROS as the ER-positive cell lines [7]. These studies suggest that estrogen induces oxidative stress, in part, by both ER-dependent and ER-independent pathways. Therefore, estrogen-induced ROS through influencing cell signaling pathways may contribute to the growth of estrogen-exposed lung cells.

Clinically, estrogen is given at a “low dose” to minimize thrombotic risk and hormone-dependent malignancies. Few in vitro and in vivo studies have studied the adverse effects of low-dose estrogen exposure. For example, high concentrations of E2 (10 *μ*M) have been shown to act as antioxidants in vitro [14], which may explain certain beneficial effects. Also, the exogenous administration of estrogen may not mimic the endogenous estrogen response because of differences in pulsatile versus continuous cell exposure. It has been argued that estrogens perhaps through antioxidant activity scavenge lipid peroxyl radicals and thus interrupting lipid peroxidation. Estrogen has been suggested to scavenge hydroxyl radicals at higher doses and inhibit superoxide radical generation [15]. Estrogen can also produce its antioxidant actions through suppressing inflammatory cytokines or modulating antioxidant enzyme status. For instance, the apoptotic oxidative effects of cytokine TNF-*α* which include ROS generation, lipid peroxidation, antioxidant enzyme consumption, and disruption of mitochondrial membrane potential may be countered by estrogen [16]. The chemical structure of estrogens contains a phenolic ring. In the presence of an oxidant-generating environment, the phenolic hydroxyl group present at the C3 position of the A ring of estrogens or catechol estrogens accepts electrons and gets oxidized by either accepting these electrons or losing a proton [12, 13]. This may help explain the antioxidant function of estrogens or estrogenic chemicals. In contrast to antioxidant effects, estrogens have been described to induce an inflammatory response with an increase of chemokines such as IL-8 [17]. On the contrary, androgens have been demonstrated to have potent anti-inflammatory effects, reducing secretion of cytokines and chemokines which are related to Th1 inflammatory response [18]. Testosterone was able to blunt the inflammatory response induced by potent proinflammatory stimuli such as TNF*α*, LPS, and activated CD4 (+) lymphocytes [19]. Hence, the counteractive effects of these two sex steroid hormones might justify the relative increased incidence of pulmonary diseases in females as compared to that in males as well as help to explain the paradoxical effects of estrogens.

Besides the dose, the capability of lung tissue to biosynthesize estrogen from circulatory testosterone by the cytochrome P-450 enzyme aromatase (CYP19) raises the question of whether a local imbalance between testosterone and E2 levels influences the development of lung disease. Lastly, cell context-specific effects may also determine whether physiological or pharmacological concentrations of E2 stimulate cell proliferation, hypertrophy, or survival of obliterative vascular lesions found in severe pulmonary arterial hypertension (PAH). Understanding the biological and biochemical differences of sex-specific lung diseases poses a major challenge in clinical research because of the predominant use of male cell lines and animal models. This has garnered the attention of NIH which has implemented an initiative to reduce sex bias in research [20]. This review will discuss the general state of knowledge of estrogens in lung disease with a focus on vessel lumen-obliterating lesions that are found in PAH. This will include a description of estrogens and xenoestrogens in lung tissue and disease, a review of sex bias in obliterative lung disease, an explanation for the sex differences in PAH, and a proposed mechanism for the formation of obliterative vascular lesions by estrogenic stress.

## 2. Estrogens and the Lung

Three major steroidal estrogens in women, estrone (E1), estradiol (E2), and estriol (E3), are produced by the ovary from cholesterol. The steroidogenesis pathway also produces ovarian androgens, specifically testosterone and androstenedione, which are aromatized to E2 by the enzyme aromatase. The cytochrome P-450 enzymes CYP1A1 and CYP1B1 metabolize E2 into two catechol estrogens, 4-hydroxyestradiol (4-OHE2) and 2-hydroxyestraidol (2-OHE2), which are further metabolized to methoxyestrogens via catechol-O-methyltransferase [12, 13]. Out of the three estrogens, E2 has the highest estrogenic activity and is the most abundant in the bloodstream during reproductive years. Women experience normal fluctuations in estrogen throughout their lifetime and in their reproductive years. Premenopausal circulating E2 levels range 40–400 pg/ml with a considerable drop after menopause to approximately 10–20 pg/ml [21]. During the menstrual cycle, E2 increases in the follicular phase (days 0–14) in the range of 40–100 pg/ml that ends with a surge of E2 ranging from 100 to 400 pg/ml on day 14. Estradiol levels are lower during the luteal phase 40–250 pg/ml and return to lower levels prior to menstruation. Men also produce estrogen, but at lower levels than women. The adult testis converts testosterone to E2 by aromatase in Leydig cells and germ cells [22]. Once in the bloodstream, estrogen can exist in two forms, bound or unbound to a protein carrier. Between 20 and 40% of circulating estradiol is bound to sex hormone-binding globulin (SHBG) which retains them in the circulation where they are considered to be inactive [23]. Estradiol that is unbound can diffuse directly through the cell membrane where it binds to estrogen receptors to regulate transcriptional processes. In addition, membrane-bound estrogen receptors mediate both genomic and nongenomic effects on target cells. Sex differences in fetal lung development and maturation of adult lung tissue have been attributed to estrogen [24]. The formation of alveoli in females depends on estrogens which modulate alveologenesis by ER*α* and ER*β* [25, 26]. The production of surfactant in the fetal lung can be increased by E2 treatment [27], which may contribute to more rapid lung maturation in female fetuses than in the male fetus [28]. Although alveolar volume and number of alveoli per unit area do not differ between male and female, males develop larger lungs with larger conducting airways in adulthood [29].

Several lung diseases are more common in women than in men; and estrogen has been implicated as a risk factor. Since the most biologically active estrogen is E2, we reviewed concentrations of E2 reported in pathological conditions of the human lung. In patients with PAH, it has been recommended to avoid pregnancy. Levels of E2 tend to rise in the bloodstream up to 7200 pg/ml during pregnancy which may exacerbate lung pathology [30]. A recent study reported a significantly higher level of circulatory E2 [42 pg/ml] and E2/testosterone ratio in men with PAH [31]. Aromatase was shown to be expressed by human pulmonary arterial smooth muscle cells in both PAH patients and controls [32]. Since E2/testosterone ratio has been considered to be correlated with aromatase activity [33], it is possible that the localized expression of aromatase may elevate E2 in the pulmonary artery. With regard to local E2 concentrations, lung tissue concentrations of 20 pg/g in non-small-cell lung cancer (NSCLC) have been reported to be 2.2-fold higher than those found in corresponding nonneoplastic lung tissues [34]. E2 concentration of 79 pg/g was reported in interstitial pneumonia (IP) which was 2.8-fold higher than in normal lung [35]. A significant immunolocalization of aromatase in IP tissues implicates a role of local metabolism in causing local estrogen overexposure in the lung. In premenopausal women, the major sources of circulatory estrogens are the ovaries. However, estrogens are produced locally in various reproductive and nonreproductive tissues in both postmenopausal women and men by enzymatic conversions of serum androgens and adrenal cortex steroids. The production of E2, the most potent estrogen, from the precursor E1 is a major conversion pathway dependent on the enzyme 17-beta-hydroxysteroid dehydrogenases (17*β*-HSDs) [36]. The enzyme CYP19A1 aromatase, mentioned previously, also catalyzes the aromatization of androstenedione to E1 and testosterone to E2. Evidence from a recent study of COPD showed that the local production of E2 in the lung had increased levels of enzymes involved in local estradiol synthesis [9]. Since chronic inflammation is a major hallmark of lung diseases such as COPD and pulmonary hypertension, we provide a summary of proinflammatory effects as it pertains to estrogen in the following section.

## 3. Proinflammatory Effects of Estrogen in the Lung

The function of estrogen in inflammation is complex because on the one hand, suppression of inflammation with increased estrogen occurs in chronic inflammatory diseases, while on the other hand, estrogen produces proinflammatory effects in some chronic autoimmune diseases. Estrogen induces proinflammatory cytokines, such as interleukin-1*β* (IL-1*β*) and tumor necrosis factor alpha (TNF-*α*), and a number of other inflammation-associated genes, which were also associated with exposure to endocrine-disrupting chemicals (EDCs) [37]. How estrogen induced inflammation may play a role in lung disease is not clear. One of the mechanisms includes inflammation-mediated oxidative stress. For example, inflammatory genes are associated among estrogens, EDCs, and several chronic diseases. Polychlorinated biphenyls (PCBs) congener 126 and congener 153 modify the following inflammation related genes: AHR, CXCL2, HMOX1, IFNG, IL6, PTGS2, SOD2, and TNF; AHR, CXCL8, HMOX1, IL1B, IL6, MMP9, NOS2, NOS3, PARP1, PTGS2, and TNF; and AHR, IFNG, IL1B, PARP1, PTGS2, and TNF. Dibutyl phthalate, diethyl-hexyl phthalate, and BPA-modified inflammation genes are AHR, CXCL8, HMOX1, IL1B, IL6, MIF, MMP9, PARP1, SOD2, TFRC, and TNF; AHR, CSF2, CXCL8, IFNG, LEP, MMP9, SOD2, and TNF; and AHR, CSF2, HMOX1, IFNG, IL1B, IL6, LEP, MIF, MMP9, NOS2, NOS3, PARP1, PTGS2, SOD2, and TNF, respectively. In addition to the direct effect of estrogen on mitochondria and the redox cycling of catechol estrogen, estrogen-induced proinflammatory cytokines, such as IL-1*β*, IL-6, and TNF-*α*, can also generate reactive oxygen and nitrogen species (RO/NS) [38]. In the pathogenesis of estrogen-dependent lung diseases, the role of IL-6 and IL-1*β* is implicated in cell proliferation, angiogenesis, and cell adhesion. The concentration of the peptide IL-1*β* seems to determine its stimulatory or inhibitory paracrine and/or autocrine signals that regulate the growth of estrogen-dependent disease [39]. IL-6 is an important cytokine involved in the pathogenesis of PAH. Clinical data showed an association between higher levels of IL-6 in PAH patients that also correlated with patient survival [40]. Furthermore, IL-6 has been shown to impact the development of pulmonary hypertension in COPD patients [41]. In the transgenic mouse model, overexpression of IL-6 resulted in obliterative neointimal lesions consisting of endothelial cells [42]. It is important to note that estrogen differentially regulates IL-6 production in various cell types; however, estrogen has been shown to stimulate IL-6 production in mice and humans [43]. Taken together, these evidences support the proinflammatory contribution of estrogens to obliterative lung lesions in chronic disease.

## 4. Xenoestrogens, Endocrine Disruptors, and the Lung

Endogenous estrogens are known to strongly regulate angiogenesis and vascular modeling by influencing the growth of both vascular endothelial and smooth muscle cells. Exogenous estrogen exposures may also be important factors to consider in sex-specific lung diseases. Pharmacological exposure to hormone replacement therapy (HRT) or oral contraceptives has been shown to exacerbate PAH [44–47], LAM [48, 49], and NSCLC [50]. There is also a growing body of evidence in support of estrogenic endocrine disruptors including occupational exposure to chlorinated solvents in PAH [51]. High levels of PCBs have been reported in human lung tissue [52]. Inhalation exposure to vapor phase PCBs was demonstrated to be even more important than ingestion under some circumstances [53]. Epidemiological studies have shown that chronic exposure to PCBs including its estrogenic congeners is associated with lung toxicity [54] and hypertension [55]. Prenatal exposures to PCBs have been associated with decreased lung function in a 20-year-old offspring [54]. Moreover, population-based studies have provided evidence that PCBs are damaging to the vascular system [56–59]. In vivo animal studies have shown that PCBs produce placental vascular lesions and trophoblastic lesions [60]. We have reported that physiological levels of E2 and estrogenic PCB153 [1 ng/ml] at a level found in human serum [0.60–1.63 ng/ml] [61] altered pulmonary endothelial as well as smooth muscle cell phenotypes [61]. PCB153's effects on both endothelial cells are even more pronounced than those on E2 with respect to vasculosphere formation and vasculogenesis. Another endocrine-disrupting chemical, 4,4′-methylenedianiline, used in the synthesis of polyurethanes has been shown to increase hyperplasia of pulmonary arteries exclusively in female rats [62]. In vitro human pulmonary smooth muscle cells were shown to proliferate when exposed to 4,4′-methylenedianiline, and this was inhibited by treatment with the estrogen receptor antagonist ICI 182,780. Another well-known xenoestrogen, bisphenol A, has been reported to enhance the development of asthma [63]. Environmentally relevant concentrations of bisphenol A have been shown to elicit proangiogenic effects in human endothelial cells [64]. Taken together, these studies suggest that exposure to xenoestrogens and/or endocrine disruptors is a potential risk factor for obliterative lung lesions.

## 5. Sex Bias in Lung Disease

### 5.1. Asthma

Gender has been shown to play a role in the diseased lung. We will summarize sex differences in major lung diseases at times highlighting how estrogens contribute to obliterative processes in the lung such as cell proliferation and migration. Female hormones in allergic disease have been extensively studied in asthma. After puberty, the prevalence of asthma is greater in girls than in boys [65]. The prevalence of asthma is greater in women than in men during early to middle adulthood [66]. Asthma is also more severe in women with a higher likelihood of death compared to that in men [67]. Modulation of lung inflammation by estrogen may partly explain this association. In asthma, inflammation enhances airway smooth muscle cell contractility, proliferation, and extracellular matrix production. Estrogens are known to modulate immune cells such as macrophages, lymphocyte, and mast cells, some of which express ERs and the estrogen membrane receptor GPR30 [68], which may contribute to smooth muscle hyperplasia that obliterates the airway.

### 5.2. Chronic Obstructive Pulmonary Disease (COPD)

Chronic obstructive pulmonary disease is a progressive disease that includes emphysema and chronic bronchitis. The incidence of COPD in women has been reported to be increasing [69]. For example, smoking is a major risk factor for COPD, but females tend to develop COPD faster than males even though they smoke less cigarettes [70]. In nonsmokers, females make up two-thirds of cases with COPD [71]. Cell proliferation has been shown to contribute to the intimal thickening of pulmonary arteries in both smokers and patients with mild COPD [72]. The early appearance of obliterative vascular lesions in COPD suggests that the pathology is not a late complication of pulmonary hypertension. Rather, the growth-promoting effects of estrogen on smooth muscle cells may be involved in the early development of COPD. Besides receptor-mediated pathways, oxidative stress from estrogen metabolism in the lung may contribute to the growth of these cells. Estrogens have been shown to be hydroxylated to catechol estrogens, and catechol estrogens participate as a substrate in cytochrome P450-catalyzed redox reactions [12, 13]. Thus, estrogen potentiation of oxidative stress may confer susceptibility of female smokers to COPD. Cystic fibrosis is a rare genetic disorder that affects both men and women and is characterized by a buildup of mucus in the lungs. This abnormal level of mucus leads to repeated, serious lung infections that over time severely damage lungs. Women have shown a higher prevalence of severe cystic fibrosis, and exacerbations coincide with estrogen peak in the menstrual cycle [73, 74]. Estrogen has been demonstrated to upregulate the MUC5B gene, a major mucin in the human airway [75]. A potential mechanism by which estrogen may exacerbate cystic fibrosis in women may be by increasing MUC5B expression.

### 5.3. Lymphangioleiomyomatosis (LAM)

Pulmonary lymphangioleiomyomatosis (LAM) is a progressive and eventually fatal disease that primarily affects premenopausal women and can be exacerbated by pregnancy [76]. Estrogen can be considered a risk factor for LAM because disease severity worsens with estrogen therapy [77]. LAM is associated with abnormal proliferation and invasion of smooth muscle cells that destroy the lung parenchyma. Small clusters of cells characterize lung lesions in LAM which are located along pulmonary bronchioles, blood vessels, and lymphatics. Clumps of LAM cells in lymph vessels lead to the thickening of the vessel wall and obliteration of the lumen. Immunohistochemical data has also shown higher levels of estrogen-synthesizing enzyme aromatase in LAM cells [78]. Lung cancer is a leading cause of cancer-related deaths in women [79]. A greater female predominance of NSCLC in both smokers and nonsmokers suggests that differences in sex hormones contribute to its pathogenesis [80]. A worse prognosis in women with lung cancer has been associated with the expression of aromatase [81]. Hence, the proproliferative effects of estrogen along with its known genotoxic effects may explain the sex bias observed in both LAM and NSCLC.

### 5.4. Pulmonary Arterial Hypertension (PAH)

Pulmonary arterial hypertension is clinically classified as group 1 in the World Health Organization (WHO) system. Uncontrolled vascular cell growth has been postulated as the major mechanism involved in PAH pathogenesis [82], which results in vessel obliteration. Most epidemiological studies have determined the effect of gender on prevalent PAH cases. Group 1 PAH includes idiopathic PAH, heritable PAH, drug- and toxin-induced PAH, and PAH-associated conditions such as connective tissue disease- (CTD-) PAH, HIV-PAH, congenital heart disease- (CHD-) PAH, and schistosomiasis. The Registry to Evaluate Early and Long-term PAH Disease Management (REVEAL) is a database used in an ongoing observational cohort study of PAH designed to enroll prevalent and/or incident patients in the United States with group 1 PAH. This study reported the highest female to male ratio of 4.1 : 1 in IPAH patients as compared to the French registry (1.9 : 1) and the National Institutes of Health registry (1.7 : 1) [83–85]. A female bias was also reported in other subcategories of group 1 PAH which include CHD-PAH (2.8 : 1), CTD-PAH (9.1 : 1), and drug-/toxin-induced PAH (5.4 : 1) [83]. We have provided a descriptive table of female to male ratios reported from these PAH registries (Table 1).

## 6. Estrogen as a Risk Factor in PAH

In human studies, pulmonary hypertension [44] and vessel lumen-obliterating lesions [46] have been associated with oral contraceptives. Hormone replacement therapy has also been associated with severe PAH in postmenopausal women [86]. While these hormone therapies contain estrogens, the contribution of estrogen to PAH has been debated because of paradoxical gender effects observed in animal models. The chronic hypoxia-induced pulmonary hypertension model showed that male rats are more susceptible than females while estrogen treatment was shown to protect against monocrotaline- (MCT-) induced pulmonary hypertension [87, 88]. In contrast, there are reports of chronic E2-induced hypoxic pulmonary hypertension in ovariectomized female rats [89–91]. The contradictory effects of E2 in the MCT-induced model may be partly due to differences in pulsatile versus continuous E2 exposure which cannot fully recapitulate what occurs in the human body. Another factor that may complicate our understanding comes from the assumption that exogenous and endogenous E2 act similarly on the pulmonary vasculature. Recently, a study has shown that reduction of endogenous E2 by ovariectomy or aromatase inhibitor treatment decreased vessel muscle-thickening or vessel-obliterating lesions [32]. This study used both the hypoxic mouse and the Sugen 5416 plus chronic hypoxia (SuHx) rat model of PAH. In the SuHx model, rats are given a single injection of the VEGF receptor blocker Sugen 5416 and exposed to hypoxia for several weeks [92]. The protection observed with the anastrazole treatment of the previous study was corroborated by a study with metformin treatment which reversed PAH and decreased pulmonary vascular remodeling via aromatase inhibition [93]. E2 treatment was reported to improve heart function in the SuHx model [94], but its effect on the development of plexiform lesions, a hallmark of human PAH reproduced in the SuHx rat model, was not reported. Further studies on the development of obliterative intimal lesions in a chronic E2-treated SuHx model would be helpful because of the previously mentioned reports of chronic E2-induced pulmonary hypertension in ovariectomized female rats.

Other rodent models of PAH have reported a female bias toward PAH. Anorectic drugs such as dexfenfluramine (Dfen) have been shown to induce PAH only in female mice [95]. Treatment of rats with 4,4′-methylenedianiline (DAPM) induced female-specific smooth muscle hyperplasia of the pulmonary vessels [62]. Genetic-based mouse models have also shown sex differences in PAH susceptibility. Female mice overexpressing calcium-binding protein S100A4/Mts1 (Mts1) were more susceptible to develop PAH and developed plexiform-like lesions [96]. In mice overexpressing the serotonin transporter (SERT), only female SERT+ mice developed PAH [97]. Since E2 treatment increased the severity of PAH in female SERT+ mice, it is plausible that estrogen is a significant risk factor for the development of PAH. Furthermore, the inhibition of obliterative vascular lesions by aromatase inhibitor anastrozole in the SuHx model supports the idea that E2 mediates its adverse effects by increasing the formation of plexiform lesions in PAH. We have provided a summary table of the discussed in vivo models that support a role of female sex and/or estrogen in PAH (Table 2).

## 7. Biological-Based Mechanisms for Sex Differences in PAH

Circulatory levels of E2 cannot explain why males who have lower levels of E2 than females develop PAH much sooner and have poorer survival. A potential explanation may lie in the different characteristics of the vascular pathology which obliterate the pulmonary artery. Blood vessels are composed of an outer layer of adventitial fibroblasts, a middle layer of smooth muscle cells (SMC), and an inner layer of endothelial cells (EC). The medial thickening of pulmonary arteries is considered the earliest pathological change in PAH [98]. Chronic hypoxia-induced PAH is characterized by medial thickening [99, 100]. Experimental data from rodent models attribute the thickening to pulmonary arterial SMC hypertrophy and extracellular matrix deposition in proximal pulmonary arteries [101–103]. In contrast, severe IPAH is characterized by clustered proliferation of EC that results in concentric obliteration of the lumina by vascular structures called plexiform lesions, which consist of the monoclonal proliferation of EC and are reported in the late stages of PAH [104]. Three-dimensional analysis of the plexiform lesion indicated that plexiform lesion is functionally important in pathogenesis because blood flow is severely obstructed along the entire length of a vessel affected by a single plexiform lesion [105]. Although both human pulmonary arterial SMC and EC have been shown to proliferate when exposed to E2 [97, 106], a difference between these cell types from PAH patients has been shown with the expression of an estrogen-synthesizing enzyme. Pulmonary arterial SMC were shown to highly express aromatase in PAH patients, but it was absent in human pulmonary arterial EC [32]. Thus, the cell context-specific difference in aromatase expression can help to explain why men have more severe PAH. Since men are ill equipped to defend against a higher body burden of E2 when compared to women, we propose that the local concentration of E2 in pulmonary arteries is higher in men with PAH. This difference in lung concentration of E2 contributes to the reported faster progression and severity of PAH in men. Although proliferative changes in pulmonary arteries play a significant role in the development of PAH, evidence from the SuHx model of PAH suggests that fibrosis is a determining factor in the poor survival rate of male patients with PAH [107]. In this study, female rats with PAH primarily showed vasculoproliferative changes in the pulmonary artery while males showed severe fibrosis in the adventitia and media of the pulmonary artery. Severe fibrosis observed in male pulmonary arteries including myocardial fibrosis was associated with impaired heart function and lower survival rates compared to females.

**Figure 1:**
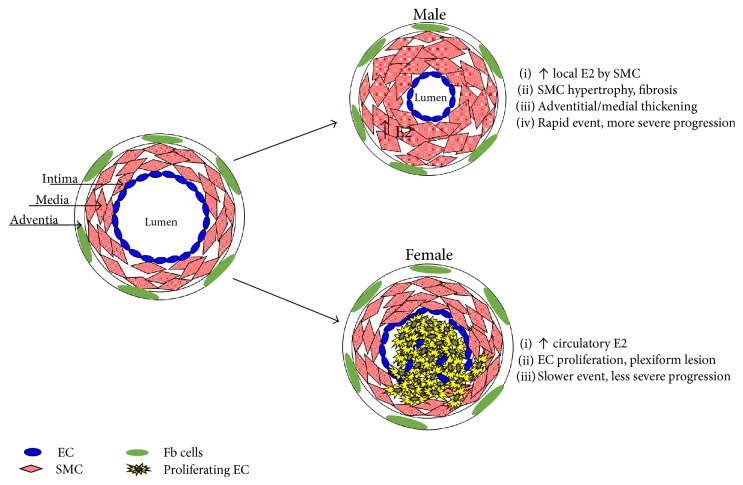
Biological sex differences in vessel obliteration.

Unlike SMC exposed to the local synthesis of E2 by aromatase, the proximity of EC to the bloodstream allows these cells to be directly exposed to circulatory E2. The possibility that estrogen is involved in the growth of EC in the plexiform lesion is suggested by the increased incidence (2.8-fold) in female PAH patients of plexiform lesions compared to their male counterparts [108]. A plausible mechanism for estrogen's involvement in plexiform lesion growth comes from evidence that infantile hemangiomas, a different type of vascular lesion, are reported with increased incidence in females with elevated levels of circulating E2 [109]. The combination of hypoxia and estrogen has been demonstrated in vitro to synergistically enhance EC proliferation [110], which we postulate to also contribute to the growth of plexiform lesions. Higher circulatory E2 may therefore explain the predominance of plexiform lesions in women with PAH because it acts directly on EC proliferation. Plexiform lesions are considered to be a late pathological event compared to the much earlier pathology of pulmonary arterial SMC hypertrophy. This suggests that the plexiform lesions in female PAH patients can take more time to obstruct the pulmonary artery unlike the more rapid hypertrophy of SMC that occurs in men, which can help to explain sex differences in disease severity. A summary scheme of the sex difference in vessel obliteration is shown in Figure 1.

## 8. Estrogen-Induced Obliterative Vascular Lesions

Vessel-obliterating lesions have been reported in female-biased lung diseases including idiopathic interstitial pneumonia [111], COPD [72], and IPAH [104]. Early appearance of obliterative vascular lesions observed in mild cases of COPD, mentioned previously, suggests that the growth of vascular lesions occurs much earlier than at the end stage of PAH. Uncontrolled vascular cell growth has been postulated as the major mechanism involved in PAH pathogenesis [82]. More specifically, the hypertrophic growth of SMC is responsible for progressive thickening of blood vessels of the lung that ends in obstruction [112]. Proliferative endothelial lesions that result from a focal budding of EC are also reported to be an aggressive cell phenotype associated with a poor prognosis in NSCLC and severe IPAH [104, 113, 114]. Despite progress in understanding IPAH, current therapy (epoprostenol and derivatives, endothelin receptor antagonists, and phosphodiesterase type 5 inhibitors) has become a major clinical barrier for the treatment of patients with end-stage IPAH. Median survival for IPAH patients in the United States was reported to be only 2.8 years without treatment [115]. Although these drugs allow clinical, functional, and hemodynamic improvements, the prognosis of patients remains poor because a critical aspect of end-stage IPAH is the continual growth of vascular lesion cells which eventually obliterate the lumen. Antiproliferative agents such as tyrosine kinase inhibitors have been investigated in IPAH; however, safety concerns have restricted the clinical application of these drugs, and therefore the need to identify new therapeutic targets has remained.

The molecular pathogenesis of vessel lumen-obliterating lesions in humans remains unknown. Largely, the focus has been on loss-of-function mutations in the BMPR2 gene observed in approximately 80% of familial PAH and in 20% of patients with sporadic PAH [116]. In addition to BMPR2, estrogen receptor signaling has been implicated to be involved in the pathogenesis of obliterative vascular lesions. However, these studies have not been consistently focused on investigating target cells (vascular lesion “initiating” cells) that are susceptible to genetic and epigenetic instability and ultimately progress into the plexiform lesion. Investigators have conveniently used either adult EC or SMC without considering the in vivo plexiform lesion histopathology. Histopathology of both human and animal model obliterative vascular lesions suggests they are multicellular and just like solid tumors that contain stem cells that may be involved in the pathogenesis of IPAH [117]. Surprisingly, there are numerous clinical and experimental data of vessel stem cells in the blood and the lungs of various forms of PAH [118]. Although several different cell types, including vascular SMC, inflammatory cells, and fibroblasts, are involved in the vasculoproliferative process, we recognize EC to be the initial site of injury. Previously, we showed that E2 treatment leads to an increase in macrophage cell proliferation and secretion of TNF-*α* [119, 120] which could contribute to vascular lesion formation via paracrine effects with other cell types in the vessel wall. Estrogen involvement in immune responses in lung diseases described previously supports an inflammatory role in PAH.

**Figure 2:**
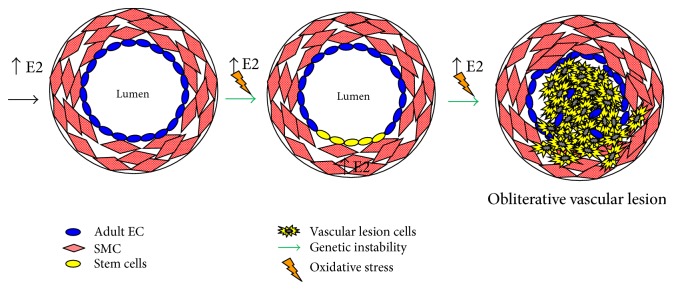
Estrogen-induced vessel lumen obliteration.

Endothelial and smooth muscle cells are directly involved in the pathology of plexiform lesions. Pulmonary arterial SMC express aromatase which allows for the local production of E2, whereas human pulmonary arterial microvascular EC do not possess this enzyme [32]. Higher aromatase activity in pulmonary arterial SMC may lead to locally produced estrogen that acts in an autocrine or paracrine manner, with possible cross talk between SMC and EC. Besides estrogen synthesis, the metabolism of E2 by another enzyme CYP1B1 may contribute to the formation of lumen-obliterating vascular lesions. CYP1B1 expression is increased in pulmonary arterial SMC from patients with IPAH [121]. Cytochrome P450 family member CYP1B1 is a key enzyme involved in the metabolism of E2 to catechol estrogens and expressed in the lung. Oxidation of E2 produces 2 catechol estrogens that, in turn, are further oxidized to the quinones, which can react with DNA resulting in depurinating adducts that can lead to mutagenesis. Genetic instability usually associated with pathological disorders and referring to a range of genetic alterations from mutations to chromosome rearrangements may contribute to the quasi-malignant vascular lesions observed in PAH patients. In support of this concept, chromosomal abnormalities and increased DNA damage have been observed in vessel lumen-obliterating lesions from PAH patients [122] and we have shown a positive correlation of oxidative DNA damage (8-OHdG) in benign and malignant vascular tissues [123]. In vivo experimental evidence in support of genotoxic damage in PAH was shown in the SERT+ model of PAH, and female SERT+ mice showed increased levels of 8-OHdG [124]. We have provided a hypothetical mechanism by which chronic estrogenic stress induces genetic instability in stem cells that progress to form the obliterative vascular lesion (Figure 2).

## 9. Conclusion

Mitogenic and genotoxic effects of estrogen may be a common pathogenic mechanism to explain the presence of obliterative lesions in lung tissue and vessels. Estrogen has been shown to promote lung disease in experimental models of PAH, lung cancer, LAM, and benign metastasizing leiomyoma (BML) [32, 80, 125, 126]. Studies have reported associations between estrogen concentrations in lung disease. Lung tissues from interstitial pneumonia are reported with 2.8-fold higher levels of E2 [35], NSCLC has high intratumoral E2 concentration associated with aromatase expression [125], and more recently, higher concentrations of E2 have been associated with the risk of PAH in men [31]. Furthermore, higher aromatase activity and circulatory E2 have been reported to increase the risk of PAH in patients with portopulmonary hypertension [127]. Based on the evidences discussed in this review, female gender bias toward obliterative lung disease may be attributed to the hormone estrogen.

Even though women have a 3-4 times higher prevalence than men of PAH, circulatory E2 levels cannot explain why men develop PAH much sooner and have poorer survival. Pulmonary arterial SMC hypertrophy that contributes to medial thickening is considered one of the earliest pathological changes observed in chronic hypoxia-induced PAH. We postulate that the severity of PAH in males is due to high local concentration of E2 produced by pulmonary arterial SMC, which leads to hypertrophy, vasoconstriction, and vessel obstruction. Since males cannot defend against a higher body burden of E2 unlike females, males succumb to a rapid and more severe progression of vascular obliteration in PAH. Females are more susceptible to develop pulmonary vascular disease characterized by obliterative hyperproliferative vascular lesions because EC are directly exposed to circulatory E2 from the bloodstream. Higher circulatory E2 found in women can therefore explain the predominance of plexiform lesions in female PAH patients. The molecular mechanisms that underlie sex differences in vessel lumen-obliterating lesions remain largely unknown, and this is a major hurdle to identifying novel sex-dependent molecular targets to treat obliterative vascular lesions. Understanding the molecular basis of this gender disparity in PAH may offer a new treatment paradigm in this devastating disease that currently has a high unmet clinical need.

Emerging evidence suggests that a local imbalance between testosterone and E2 levels influences the development of lung disease in COPD and PAH. In light of this information, we propose that novel therapies targeted against local tissue production of estrogen may be of clinical benefit and lead to novel therapeutic strategies in treating estrogen-dependent lung diseases. The activation of the farnesoid X receptor (FXR) has been reported to inhibit aromatase at the level of mRNA, protein, and enzymatic activity [128] and represents a novel therapeutic mechanism to reduce local tissue estrogen production in the lung. The potential inhibitory effect of FXR on aromatase is significant because a new class of drugs (FXR agonist, such as obeticholic acid (OCA)) was recently shown to prevent monocrotaline-induced PAH [129]. Similar cardiopulmonary protective effects of OCA treatment have been demonstrated also in blemycin-induced pulmonary fibrosis [130]. FXR activation by treatment with OCA was shown to protect against bleomycin-induced lung damage by suppressing epithelial-to-mesenchymal transition (EMT), inflammation, and collagen deposition. This may be of major benefit in the treatment of PAH. Endothelial-to-mesenchymal transition (EndMT), a process similar to EMT, has been implicated to contribute to obliterative vascular remodeling in idiopathic PAH [131]. Furthermore, the release of cytokines IL-1*β*, IL-6, TNF-alpha, and IL-10 by macrophages present in pulmonary lesions are suggested to play an important role in the pathogenesis of PAH [40]. Since FXR activation was shown to suppress EMT as well as cause a dose-dependent reduction of proinflammatory cytokines, the FXR class of drugs is highly innovative therapeutic agents for the treatment of estrogen-dependent obliterative lung diseases including PAH.

## Figures and Tables

**Table 1:** Summary of PAH registry female to male ratios.

Registry	Time	Cohort	Number of patients	Female : male ratio	References
REVEAL	2006-2007	Mean age 53 yr IPAH, HPAH, APAH, drug-/toxin-induced PAH	2525	4.1 : 1 IPAH 3.8 : 1 APAH 5.4 : 1 drug-/toxin-induced PAH	[83]
French	2002-2003	Mean age 50 yr IPAH, HPAH, drug-/toxin-induced PAH	674	1.9 : 1	[84]
NIH	1981–1985	Mean age 36 yr IPAH, HPAH	187	1.7 : 1	[85]

IPAH: Idiopathic PAH; HPAH: Heritable PAH; APAH: Associated PAH.

**Table 2:** Models of PAH that support female sex bias and/or detrimental effect of estrogen.

Model	Species	Findings	References
Chronic Hx + E2	Rat	Female develops hypoxic pulmonary hypertension; E2 detrimental	[89, 90]
SuHx	Rat, mouse	Male and female develop PAH; aromatase inhibition protective	[32, 92, 93]
Dexfenfluramine	Mouse	Female only develops PAH; Ovx protective	[95]
4,4′-Methylenedianiline	Rat	Female only develops PAH	[62]
Mts1+	Mouse	PAH in female > male Ovx protective	[96]
SERT+	Mouse	Female only develops PAH Ovx protective; E2 detrimental	[97]

Hx: Hypoxia; E2: 17*β*-Estradiol; SuHx: Sugen 5416 plus hypoxia; Mts1+: Overexpression of calcium-binding protein S100A4/Mts1; SERT+: Overexpression of serotonin transporter; Ovx: Ovariectomized.
